# In Vitro Assessment of Antimicrobial, Antioxidant, and Cytotoxic Properties of Saccharin–Tetrazolyl and –Thiadiazolyl Derivatives: The Simple Dependence of the pH Value on Antimicrobial Activity

**DOI:** 10.3390/ph12040167

**Published:** 2019-11-12

**Authors:** Luís M. T. Frija, Epole Ntungwe, Przemysław Sitarek, Joana M. Andrade, Monika Toma, Tomasz Śliwiński, Lília Cabral, M. Lurdes S. Cristiano, Patrícia Rijo, Armando J. L. Pombeiro

**Affiliations:** 1 Centro de Química Estrutural (CQE), Instituto Superior Técnico, Universidade de Lisboa, Av. Rovisco Pais, 1049-001 Lisboa, Portugal; pombeiro@ist.utl.pt; 2 CBIOS—Research Center for Health Sciences & Technologies, ULusófona de Humanidades e Tecnologias, Campo Grande 376, 1749-024 Lisboa, Portugal; ntungweepolengolle@yahoo.com (E.N.); p5319@ulusofona.pt (J.M.A.); 3 Department of Biology and Pharmaceutical Botany, Medical University of Lodz, Muszyńskiego Street 1, 90-151 Łódź, Poland; przemyslaw.sitarek@umed.lodz.pl; 4 Laboratory of Medical Genetics, Faculty of Biology and Environmental Protection, University of Lodz, 90-151 Lodz, Poland; monika.toma@biol.uni.lodz.pl (M.T.); tomasz.sliwinski@biol.uni.lodz.pl (T.Ś.); 5 Department of Chemistry and Pharmacy (FCT) and Center of Marine Sciences (CCMar), Universidade do Algarve, P-8005-039 Faro, Portugal; liliacabral80@gmail.com (L.C.); mcristi@ualg.pt (M.L.S.C.); 6 iMed.ULisboa - Research Institute for Medicines, Faculdade de Farmácia, Universidade de Lisboa, Av. Prof. Gama Pinto, 1649-003 Lisboa, Portugal

**Keywords:** saccharin, tetrazole, 1,3,4-thiadiazole, H7PX glioma cells, antimicrobial screening, antioxidant capacity

## Abstract

The antimicrobial, antioxidant, and cytotoxic activities of a series of saccharin–tetrazolyl and –thiadiazolyl analogs were examined. The assessment of the antimicrobial properties of the referred-to molecules was completed through an evaluation of minimum inhibitory concentration (MIC) and minimum bactericidal concentration (MBC) values against Gram-positive and Gram-negative bacteria and yeasts. Scrutiny of the MIC and MBC values of the compounds at pH 4.0, 7.0, and 9.0 against four Gram-positive strains revealed high values for both the MIC and MBC at pH 4.0 (ranging from 0.98 to 125 µg/mL) and moderate values at pH 7.0 and 9.0, exposing strong antimicrobial activities in an acidic medium. An antioxidant activity analysis of the molecules was performed by using the DPPH (2,2-diphenyl-1-picrylhydrazyl) method, which showed high activity for the TSMT (*N*-(1-methyl-2*H*-tetrazol-5-yl)-*N*-(1,1-dioxo-1,2-benzisothiazol-3-yl) amine, **7**) derivative (90.29% compared to a butylated hydroxytoluene positive control of 61.96%). Besides, the general toxicity of the saccharin analogs was evaluated in an *Artemia salina* model, which displayed insignificant toxicity values. In turn, upon an assessment of cell viability, all of the compounds were found to be nontoxic in range concentrations of 0–100 µg/mL in H7PX glioma cells. The tested molecules have inspiring antimicrobial and antioxidant properties that represent potential core structures in the design of new drugs for the treatment of infectious diseases.

## 1. Introduction

**Figure 1:**
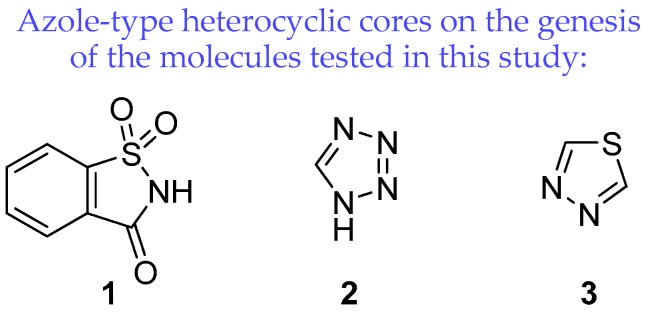
Structures of 1,2-benzisothiazole-3-one 1,1-dioxide (**1**, saccharin), 1*H*-tetrazole (**2**), and 1,3,4-thiadiazole (**3**).

In 1878, 1,2-benzisothiazole-3-one 1,1-dioxide (**1**, Figure 1), commercially known as saccharin, was discovered accidentally by Fahlberg during an investigation of the oxidation of *o*-toluenesulfonamide [1,2]: it was published by Remsen and Fahlberg one year later [3]. For more than a century, saccharin has been commonly used as a noncaloric artificial sweetener in the form of its water-soluble salts (mainly sodium, ammonium, and calcium), and it is the principal sweetening component of diabetic diets. For about three decades (since reports on carcinogenicity in laboratory animals were published), the debate on its toxicity to humans has not reached a consensus [4,5,6]. Numerous *N*-substituted derivatives of saccharin have been assessed for in vitro biological activity [7,8,9,10]. For example, first-row transition metal saccharinates as well as dioxovanadium(VI), dioxouranium(VI), and cerium(IV) saccharinates have been classified as protease inhibitors, and several metal(II) saccharinates have displayed superoxide dismutase-like activity [11]. Besides, structure–activity relationship studies have shown that the saccharin scaffold is an effective element for the development of inhibitors of human leukocyte clastase (HLE), cathepsin G (Cat G), and proteinase 3 (PR3), as well as antimycobacterium and central nervous system agents [9,12,13,14]. Recently, different saccharin-based antagonists have been recognized for their interferon-signaling pathways, showing antitumor activity through the inhibition of cancer-related isoforms in humans [15]. It should also be noted that the first non-benzoannelated 4-amino-2,3-dihydroisothiazole 1,1-dioxide, which lacks a 3-oxo group, has been described and shows anti-HIV-1 activity. Additionally, saccharin and isothiazolyl derivatives have been used in agriculture as herbicides, fungicides, and pesticides [16].

Tetrazole (CN_4_H_2_) and its derivatives have attracted much attention as well due to their practical applications. The tetrazolic acid fragment –CN_4_H has acidity similar to the carboxylic acid group –CO_2_H and is almost allosteric with it, but it is metabolically more stable at the physiologic pH [17]. Hence, synthetic methodologies leading to the replacement of –CO_2_H groups by –CN_4_H groups in biologically active molecules are of major relevance [18]. Indeed, the number of patent claims and publications related to medicinal uses of tetrazolyl derivatives continues to grow rapidly and cover a wide range of applications: tetrazoles have been found, for instance, in compounds with antihypertensive, antiasthmatic, antitubercular, antimalarial, and antibiotic activity [19,20,21,22]. Several tetrazole derivatives have shown potential as anticonvulsants and anticancer and anti-HIV-1 drugs [23,24,25]. Tetrazoles have also had important applications in agriculture as plant growth regulators, herbicides, fungicides [26], and stabilizers in photography and photoimaging [27]. Due to the high enthalpy of formation, tetrazole decomposition results in the liberation of two nitrogen molecules and a significant amount of energy. Therefore, several tetrazole derivatives have been explored as explosives, propellant components for missiles, and gas generators for airbags (applicable to the automobile industry) [28]. In addition, various tetrazole-based compounds have good coordination properties and are able to form stable complexes with several metal ions. This ability is successfully used in analytical chemistry for the removal of heavy metal ions from liquids and in chemical systems formulated for metal protection against corrosion [29]. Many physical, chemical, physicochemical, and biological properties of tetrazoles are closely related to their ability to behave as acids and bases. In the tetrazole ring, the four nitrogen atoms connected in succession are able to be involved in proteolytic processes. This heterocyclic system is unusual in structure and unique in terms of acid–base characteristics.

In line with what is mentioned above for saccharin and tetrazole derivatives, the 1,3,4-thiadiazole scaffold represents an important class of core structures that are of great interest mainly because of their various biological activities and respective therapeutic applications. The 1,3,4-thiadiazole ring is a very weak base, possesses relatively high aromaticity, and is moderately stable in aqueous acid solutions although it is vulnerable to ring cleavage with an aqueous base [30]. Besides, this heterocyclic ring is very electron-deficient due to the electron-withdrawing effect of the nitrogen atoms and is relatively inert toward electrophilic substitution but susceptible to nucleophilic attack, whereas when substitutions are introduced into the 2′ or 5′ position of this ring, it is highly activated and readily reacts to produce varied derivatives [31]. To some extent, these specific properties lead to the application of 1,3,4-thiadiazole derivatives in pharmaceutical, agricultural, and material chemistry. Therefore, several 1,3,4-thiadiazole-based compounds display a broad spectrum of biological activities, such as antimicrobial [32], antituberculosis [33], antioxidant [34], anti-inflammatory [35], anticonvulsant [36], antidepressant, anxiolytic [37], antihypertensive [38], anticancer [39], and antifungal activity [40]. The most prominent thiadiazole derivative is possibly the acetazolamide [*N*-(5-sulfamoyl-1,3,4-thiadiazol-2-yl)acetamide], a very well-known carbonic anhydrase inhibitor that is used in the treatment of glaucoma [41], high-altitude illness [42], epileptic seizures [43], idiopathic intracranial hypertension [44], hemiplegic migraine [45], cystinuria [46], obstructive sleep apnea [47], and congenital myasthenic syndromes [48].

The search for new molecular entities, whether natural or synthetic, with relevant pharmacological activities for effective applications in medical practices continues to be a hot topic in health science as well as in science as a whole. This permanent search is well documented in the immense scientific literature and is mainly supported by the fact that many pathogenic organisms are able to develop mechanisms of resistance to high-activity medicines in their early lives [49,50,51,52,53,54]. In this context, and given our prior interest in the synthesis, reactivity, and bioactivity of tetrazole and thiazole derivatives, our collaborative interest was piqued by the great potential of these heterocycles for medical applications. Herein, we report on the antimicrobial, antioxidant, and cytotoxic activities of four mixed-azole compounds of benzisothiazole–tetrazolyl and benzisothiazole–thiadiazolyl.

## 2. Results and Discussion

### 2.1. Chemistry

**Scheme 1:**
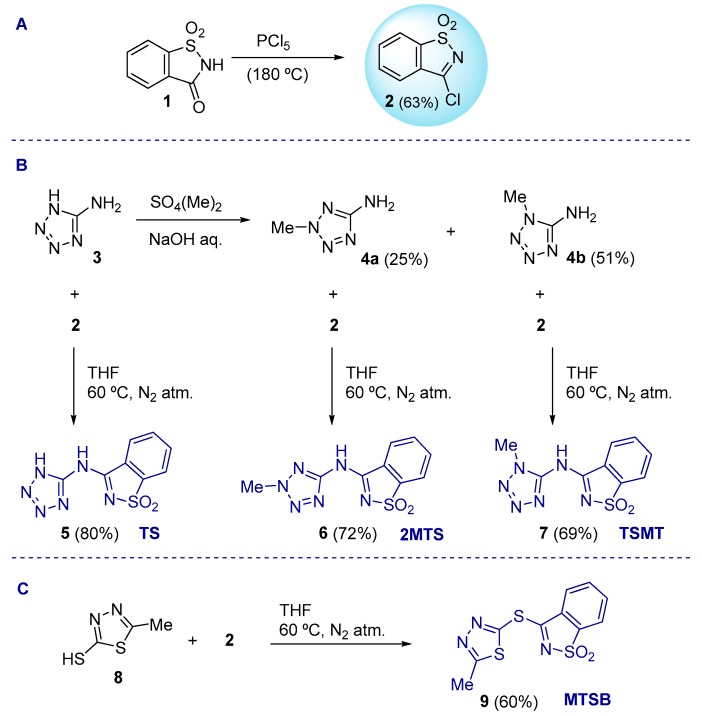
Synthesis of saccharin–tetrazolyl (TS, 2MTS, and TSMT) and saccharin–thiadiazolyl (MTSB) derivatives.

The 3-chloro-1,2-benzisothiazole 1,1-dioxide (**2**), one of the strategic building blocks for the synthesis of the studied molecules, was first prepared through the halogenation of saccharin (**1**), as previously described (Scheme 1 (A)) [55]. The three saccharin–tetrazolyl analogs (TS (**5**), 2MTS (**6**), and TSMT (**7**)) were synthetized through a combination of the amino-tetrazoles **3**, **4a**, and **4b** with **2**, following a nucleophilic substitution reaction of the chloride anion by the amine functionality (Scheme 1 (B)). Similarly, compound **9** (MTSB) was prepared by coupling **2** and 5-methyl-1,3,4-thiadiazole-2-thiol (**8**) (Scheme 1 (C)). All of the reactions proceeded smoothly under experimental protocols originally developed by us [56,57,58,59], affording crystalline products in reasonable to very good yields.

### 2.2. Biological Assays

#### 2.2.1. Antimicrobial Activity

The assessment of the antibacterial properties of compounds TSMT, MTSB, TS, and 2MTS was determined through minimum inhibitory concentration (MIC) and minimum bactericidal concentration (MBC) values against Gram-positive (*Staphylococcus aureus* and *Enterococcus faecalis*), Gram-negative (*Pseudomonas aeruginosa* and *Escherichia coli*), and yeast (*Saccharomyces cerevisiae* and *Candida albicans*) strains obtained from the American Type Culture Collection (ATCC) (Table 1). The MIC value corresponding to the lowest concentration at which no visible growth was observed was assessed by the microdilution method [60]. For MBC evaluation, the bacterial suspension in the wells was homogenized, serially diluted, spread in triplicate on appropriate medium, and incubated at 37 °C. All compounds and respective positive controls were tested at the same concentration of 500 µg/mL.

The results of the antimicrobial assay showed that the compounds tested were bacteriostatic. This was concluded because the MBC values were much higher than the MIC values (see Table 1). The compounds were more active against the Gram-negative *P. aeruginosa* and the Gram-positive *S. aureus* bacteria. However, it seemed that the MIC and MBC values were more similar against *E. coli*. Additionally, antimicrobial tests were performed at different pH values (pH 4.0, 7.0, and 9.0), also using the microdilution method (see Table 2, Table 3 and Table 4). The microorganisms used in these tests were chosen based on the initial results from the antimicrobial screening of Gram-positive bacteria and comprised four Gram-positive strains, namely *S. aureus* CIP6538, *E. faecalis* ATCC 29212, methicillin-resistant *S. aureus* CIP106760 (MRSA), and vancomycin-resistant *E. faecalis* ATCC51299 (VRE).

The results of the antimicrobial assays at the different pH values showed that lowering the medium pH to 4.0 had a positive effect on the compounds MTSB, TS, and 2MTS against the methicillin-resistant *S. aureus* CIP106760 (MRSA). Overall, it was attested that MTSB at pH 4.0 was the most active derivative against all Gram-positive strains. It should be noted that MTSB was the sole compound comprising the thiadiazole function, and presumably, the different activity must be correlated with the type of heterocycle ring. The results for pH 7.0 and 9.0 did not provide better results than those previously obtained.

In terms of pH-dependent antimicrobial mechanisms, it should be emphasized that several antimicrobial peptides (AMPs) have increasingly been reported as potent antibiotics that utilize pH-dependent antimicrobial mechanisms [61]. Some of these antibiotics display high pH optima related to their antimicrobial activity and show activity against microbes that present low pH optima, which reflects the acidic pH generally found at their sites of action, namely the skin. This effect should be comparable to our compounds and could be the explanation for the high antimicrobial activity of our compounds at low pH. Several pH-dependent AMPs and other antimicrobial proteins have been developed for medical purposes and have successfully gone through clinical trials, namely kappacins, LL-37, histatins, lactoferrin, and their derivatives. The major examples of the therapeutic applications of these antimicrobial compounds include wound healing as well as the treatment of multiple infections. Generally, such applications involve topical administration, a source of novel biologically active agents that could aid in the fulfilment of the urgent need for alternatives to conventional antibiotics, helping to avert a return to the pre-antibiotic era: our compounds could be a key development.

#### 2.2.2. Antioxidant Activity

**Figure 2:**
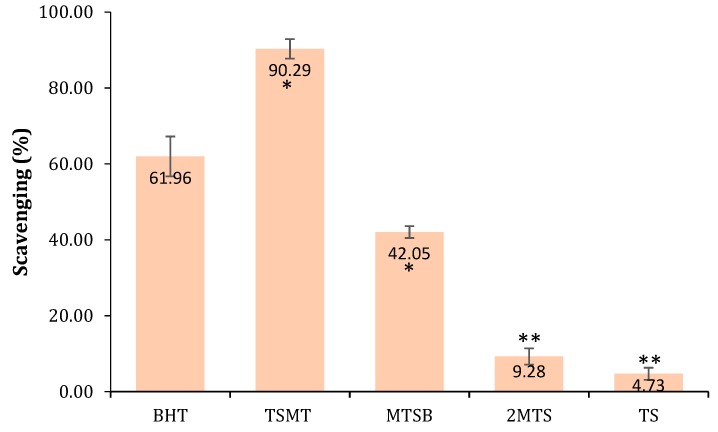
Antioxidant activity tested at a concentration of 10 μg/mL. The mean value ± SD was calculated from three independent experiments and compared to butylated hydroxytoluene (BHT) (∗ *p* < 0.05; ∗∗ *p* < 0.001).

The antioxidant activity of the studied compounds was evaluated using a DPPH assay, which evaluates the potential of test samples to quench DPPH radicals via hydrogen-donating ability. The antioxidant agents convert DPPH into a stable diamagnetic molecule, 1-1diphenyl-2-picryl hydrazine, through electron or hydrogen transfers. A color change from purple to yellow indicates the increasing radical scavenging activity of the test compounds. Herein, it was observed that TSMT and MTSB derivatives possessed a high free radical scavenging ability (see Figure 2). These two molecules had the highest reducing power, indicating that they were good electron/hydrogen donors and could prevent oxidative stress.

#### 2.2.3. Brine Shrimp Lethality Bioassay (General Toxicity)

**Figure 3:**
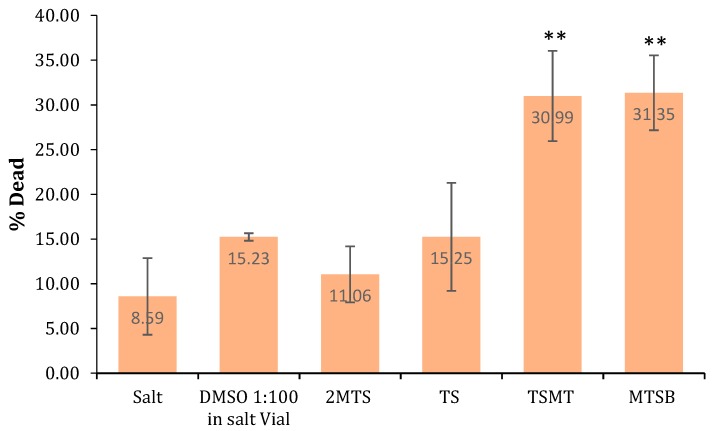
General toxicity screening at a concentration of 10 ppm using the *Artemia salina* test. The mean value ± SD was calculated from three independent experiments and compared to salt (∗∗ *p* < 0.001).

The *Artemia salina* test is a known, simple, fast, and low-cost test and was used in this investigation to check the general toxicity of the compounds. As a general rule, it was observed that all of the compounds exhibited low toxicity in the *Artemia salina* model (Figure 3). Nevertheless, it should be emphasized that the MTSB and TSMT derivatives presented with higher toxicity, making them potential lead therapeutic agents, and thus they must further be tested using different cell- and microorganism-based assays.

#### 2.2.4. Cell Viability after Treatment with MTSB, TS, TSMS, and 2MTS in H7PX Glioma Cells (IV Grade)

**Figure 4:**
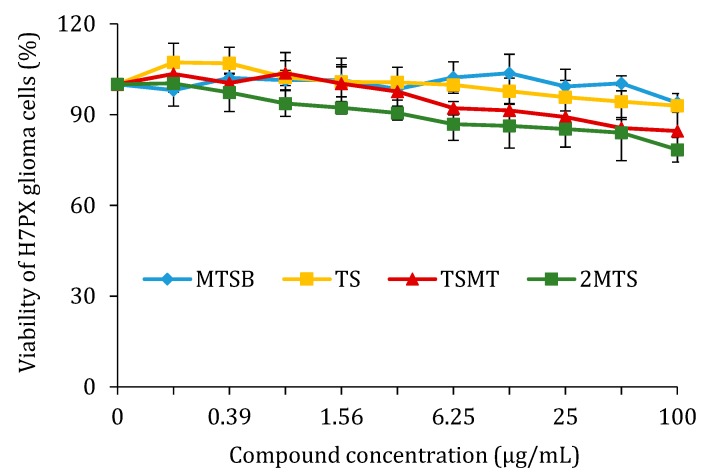
Effect of MTSB, TS, TSMT, and 2MTS treatment on the viability of H7PX glioma cells after 24 h of incubation (data are reported as means ± SD of three determinations).

In the course of this study, H7PX cells were treated with a concentration range of 0–100 µg/mL of the four compounds (MTSB, TS, TSMS, and 2MTS) over 24 h, after which the percentage of cell viability was determined by an MTT assay. It was demonstrated that none of the tested compounds reduced the viability of human H7PX cells in the stated range of concentration (0–100 µg/mL) after 24 h (Figure 4). None of the compounds’ IC_50_ values were reached. Similar effects were observed with 48 h of incubation (data not shown).

## 3. Experimental Section

### 3.1. Chemistry

#### 3.1.1. General

Unless indicated otherwise, solvents and starting materials were obtained from Sigma. All chemicals used were of reagent grade without further purification before use. Column chromatography was performed using silica gel 60 MN, and aluminum-backed silica gel Merck 60 F254 plates were used for analytical thin-layer chromatography (TLC). Melting points were recorded and are uncorrected. ^1^H and ^13^C NMR spectra were recorded at room temperature on a Bruker Avance II 400 (UltraShield™ Magnet, Billerica, MA, USA) spectrometer operating at 400 MHz (^1^H) and 101 MHz (^13^C). The chemical shifts are reported in ppm using TMS (tetramethylsilane) as an internal standard. Carbon, hydrogen, and nitrogen elemental analyses were carried out by the Microanalytical Service of the Instituto Superior Técnico—University of Lisbon. FT-IR spectra (4000–400 cm^–1^) were recorded on a VERTEX 70 (Bruker, Billerica, MA, USA) spectrometer using KBr pellets. Mass spectra were obtained on a VG 7070E mass spectrometer through electron ionization (EI) at 70 eV.

#### 3.1.2. Synthetic Protocols

The synthesis of 3-chloro-1,2-benzisothiazole-1,1-dioxide (**2**), 2-methyl-(2*H*)-tetrazole-5-amine (**4a**), 1-methyl-(1*H*)-tetrazole-5-amine (**4b**), *N*-(1*H*-tetrazol-5-yl)-*N*-(1,1-dioxo-1,2-benzisothiazol-3-yl) amine (**5**) (TS), *N*-(2-methyl-2*H*-tetrazol-5-yl)-*N*-(1,1-dioxo-1,2-benzisothiazol-3-yl) amine (**6**) (2MTS), *N*-(1-methyl-2*H*-tetrazol-5-yl)-*N*-(1,1-dioxo-1,2-benzisothiazol-3-yl) amine (**7**) (TSMT), and 3-[(5-methyl-1,3,4-thiadiazol-2-yl)sulfanyl]-1,2-benzisothiazole 1,1-dioxide (**9**) (MTSB) was carried out as previously described [55,56,58,59].

### 3.2. Biologic Activities

#### 3.2.1. Antioxidant Activity (DPPH Method)

The antioxidant activity of all the compounds was measured by the DPPH method, as described by Rijo et al. [62]. Accordingly, a mixture containing 10 μL of sample and 990 μL of DPPH solution (0.002% in methanol) was incubated for 30 min at room temperature followed by absorbance measurements at 517 nm against the corresponding blank sample. The antioxidant activity of each compound was calculated using Equation (1). *AA* denotes the antioxidant activity, *A_DPPH_* is the absorption of DPPH against the blank, and *A_sample_* represents the absorption of the compound or control against the blank. All tests were carried out in triplicate at a sample concentration of 10 µg/mL. The reference standard used for this procedure was butylated hydroxytoluene (BHT) in the same conditions as the samples:

(1) $$\mathrm{AA}\;=\;\frac{A_{\mathrm{DPPH}}\;-A_{\mathrm{Sample}}}{A_{\mathrm{DPPH}}}\times 100\%,$$

#### 3.2.2. Brine Shrimp Lethality Bioassay (General Toxicity)

The general toxicity of the compounds was evaluated by the use of a test of lethality to *Artemia salina* (brine shrimp) [63]. Concentrations of 10 ppm of each sample were tested. The number of dead larvae was recorded after 24 h and was used to calculate the lethal concentration (%) according to Equation (2) (where *Total_A. salina_* = the total number of larvae in the assay and *Alive_A. salina_* = the number of alive *A. salina* larvae in the assay):

(2) $$\mathrm{Letal}\;\mathrm{concentration}\;=\;\frac{Total_{A.\;salina}-Alive_{A.\;salina}}{Total_{A.\;salina}}\;\times 100\%,$$

#### 3.2.3. Cell Culture

An H7PX primary glioblastoma cell line was cultivated in DMEM (Biowest, Nuaollé, France) supplemented with 10% FBS (Euroclone, Pero, Italy), 100 IU/mL penicillin (Sigma-Aldrich, Saint Louis, MO, USA), 100 μg/mL streptomycin (Sigma-Aldrich, Saint Louis, MO, USA) and 50 μg/mL gentamicin (Biowest, Nuaollé, France) in a humidified atmosphere (5% CO_2_, 37 °C).

#### 3.2.4. In Vitro Cell Viability by MTT Assay

An MTT (3-(4,5-dimethylthiazol-2-yl)-2,5-diphenyl tetrazolium bromide) assay was employed to measure the viability of H7PX cells (glioma cells in IV grade derived from patient) treated with different concentrations of TS (**5**), 2MTS (**6**), TSMT (**7**), or MTSB (**9**). Cells were seeded at 1 × 10^4^ cells per well in 96-well culture plates and were left overnight before treatments for attachment. Subsequently, the cells were incubated for 24 h with all compounds over a range of concentrations: 0.0 (control), 0.39, 0.78, 1.56, 3.13, 6.25, 12.5, 25, 50, and 100 µg/mL. Following this, the cells were incubated with 0.5 mg/mL of MTT at 37 °C for 1.5 h. After that, the MTT was carefully removed, and DMSO (100 μL) was added to each well and vortexed at low speed for 5 min to fully dissolve the formazan crystals. Absorbance was measured at 570 nm with a reference at 630 nm using a Bio-Tek Synergy HT Microplate Reader (Bio-Tek Instruments, Winooski, VT, USA). All experiments were repeated in triplicate. Cell viability was expressed as a percentage relative to the untreated cells, which was defined as 100%. The study was approved by the Ethical Commission of the Medical University of Lodz, and informed consent was obtained from the patients (Nr. RNN/194/12/KE).

#### 3.2.5. Antimicrobial Activity

Microorganisms and growth conditions: The strains used in this study comprised *Staphylococcus aureus* (ATCC 25,923 and CIP 106760), *Enterococcus faecalis* (ATCC 51,299 and ATCC29212), *Escherichia coli* ATCC 25922, *Pseudomonas aeruginosa* ATCC 27,853, and the yeasts *Candida albicans* ATCC 10,231 and *Saccharomyces cerevisiae* ATCC 2601. All bacteria were grown at 37 °C in Mueller–Hinton broth (Biokar Diagnostics, Beauvais, France), and the yeasts were grown in Sabouraud dextrose agar (Biokar Diagnostics, Allone, France).

Microdilution method (MIC determination): The minimum inhibitory concentrations (MICs) of the compounds (dissolved in the respective pH aqueous solutions) were evaluated using a twofold serial broth microdilution assay (CLSI, 2011) in Mueller–Hinton broth (MHB, Biokar Diagnostics, Beauvais, France). Overnight cultures were diluted in MHB with increasing concentrations of each compound (in µM). Vancomycin (VAN), norfloxacin (NOR), and nystatin (NYS) were used as positive controls for Gram-positive bacteria, Gram-negative bacteria, and yeasts, respectively. The negative control for the aqueous solutions at different pH values showed no inhibition growth. The cultures were incubated for 24 h at 37 °C, and Optical Density at 620 nm was measured using a Microplate Reader (Thermo Scientific Multiskan FC, Loughborough, UK). Assays were carried out in triplicate for each tested microorganism.

Minimum bactericidal concentration (MBC) assessment: To define the minimum bactericidal concentration (MBC) for each set of wells in the MIC determination, a loopful of agar was collected from the wells without any growth and inoculated on sterile Mueller–Hilton medium broth (for bacteria) through streaking. Plates inoculated with bacteria were incubated at 37 °C for 24 h. After incubation, the lowest concentration was noted as the MBC (for bacteria) at which no visible growth was observed.

#### 3.2.6. Statistical Analysis

The values in this study are expressed as means ± SD. The Shapiro–Wilk test was used for verification of the normality of the data. Statistical differences were determined by one-way ANOVA. The results were analyzed using STATISTICA 12.0 software (StatSoft, Tulsa, OK, USA). Differences of *p* < 0.05 were considered statistically significant. 

## 4. Conclusions

The antimicrobial, antioxidant, and cytotoxic activities of three saccharin–tetrazolyl (TS, TSMT, and 2MTS) derivatives and one saccharin–thiadiazolyl (MTSB) derivative were addressed throughout this investigation. The antimicrobial activity of the synthesized compounds was evaluated against a series of Gram-positive and Gram-negative bacteria and yeast strains. An evaluation of the MIC and MBC values of the four derivatives was completed at pH 4.0, 7.0, and 9.0 against four Gram-positive strains (*S. aureus*, *E. faecalis*, *S. aureus* (MRSA), and *E. faecalis* (VRE)), showing high values for the MIC and MBC at pH 4.0 (ranging from 3.42 to 473.0 µM). It was attested that the derivative MTSB, the sole compound comprising the thiadiazole function, was the most active against all of the considered Gram-positive strains at pH 4.0. 

In addition, the antioxidant activity of the compounds (calculated by using the DPPH method) was the highest value for TSMT (90.29% compared to the BHT positive control of 61.96%). Finally, we demonstrated for the first time that the TS, TSMT, 2MTS, and MTSB compounds did not show in vitro cytotoxic effects on H7PX glioma cells. 

The present study exposed the influence of the pH of the medium on the antimicrobial activity of the TS, TSMT, 2MTS, and MTSB compounds, which is similar to well-described antimicrobial peptide antibiotics. Therefore, the use of these kinds of molecules to produce new antibiotics should be considered in the future, although further studies are needed to confirm this hypothesis.

## Figures and Tables

**Table 1:** Minimum inhibitory concentration (MIC) and minimum bactericidal concentration (MBC) values of TSMT, MTSB, TS, and 2MTS (obtained through the microdilution method against Gram-positive bacteria, Gram-negative bacteria, and yeast strains (in µM)).

	*E. faecalis*		*S. aureus*		*P. aeruginosa*		*E. coli*		*S. cerevisiae*		*C. albicans*	
Sample	MIC	MBC	MIC	MBC	MIC	MBC	MIC	MBC	MIC	MBC	MIC	MBC
TSMT	236.5	1892.1	118.3	1892.1	118.3	946.0	236.5	473.0	473.0	946.0	236.5	1892.1
MTSB	219.0	1752.1	109.5	1752.1	109.5	876.1	219.0	438.0	438.0	876.1	219.0	1752.1
TS	249.8	1998.1	124.9	1998.1	124.9	999.0	249.8	499.5	499.5	999.0	249.8	1998.1
2MTS	236.5	1892.1	118.3	1892.1	118.3	946.0	236.5	473.0	473.0	946.0	236.5	1892.1
Positivecontrol	1.95	nt	1.95	nt	0.977	nt	0.488	nt	15.6	nt	7.81	nt
	VAN		VAN		NOR		NOR		NYS		NYS	

VAN: vancomycin; NOR: norfloxacin; NYS: nystatin; nt: not tested. Data are the median values of at least three replicates.

**Table 2:** MIC and MBC values of TSMT, MTSB, TS, and 2MTS (obtained through the microdilution method against Gram-positive bacteria at pH 4.0).

	*S. aureus CIP6538*		*E. faecalis ATCC51299* *(VRE)*		*S. aureus CIP106760* *(MRSA)*		*E. faecalis 29212*	
Sample	MIC	MBC	MIC	MBC	MIC	MBC	MIC	MBC
TSMT	473.0	1892.1	236.5	1892.1	236.5	473.0	236.5	1892.1
MTSB	13.7	109.5	13.7	109.5	3.42	1.71	3.42	27.4
TS	62.5	499.5	31.2	249.8	3.90	1.95	7.80	62.5
2MTS	29.6	236.5	14.8	118.3	3.70	1.85	473.0	1892.1
Positive control	1.95	nt	1.95	nt	0.977	nt	0.488	nt
	VAN		VAN		NOR		NOR	

VAN: vancomycin; NOR: norfloxacin; NYS: nystatin; nt: not tested. Data are the median values of at least three replicates.

**Table 3:** MIC and MBC values of TSMT, MTSB, TS, and 2MTS (obtained through the microdilution method against Gram-positive bacteria at pH 7.0).

	*S. aureus CIP6538*		*E. faecalis ATCC51299* *(VRE)*		*S. aureus CIP106760* *(MRSA)*		*E. faecalis 29212*	
Sample	MIC	MBC	MIC	MBC	MIC	MBC	MIC	MBC
TSMT	473.0	1892.1	236.5	1892.1	473.0	1892.1	946.0	1892.1
MTSB	438.0	1752.1	219.0	1752.1	438.0	1752.1	876.1	1752.1
TS	499.5	1998.1	249.8	1998.1	499.5	1998.1	999.0	1998.1
2MTS	473.0	1892.1	236.5	1892.1	473.0	1892.1	946.0	1892.1
Positive control	1.95	nt	1.95	nt	0.977	nt	0.488	nt
	VAN		VAN		NOR		NOR	

VAN: vancomycin; NOR: norfloxacin; NYS: nystatin; nt: not tested. Data are the median values of at least three replicates.

**Table 4:** MIC and MBC values of TSMT, MTSB, TS, and 2MTS (obtained through the microdilution method against Gram-positive bacteria at pH 9.0).

	*S. aureus CIP6538*		*E. faecalis ATCC51299* *(VRE)*		*S. aureus CIP106760* *(MRSA)*		*E. faecalis 29212*	
Sample	MIC	MBC	MIC	MBC	MIC	MBC	MIC	MBC
TSMT	946.0	1892.1	473.0	1892.1	473.0	1892.1	473.0	1892.1
MTSB	438.0	1752.1	219.0	1752.1	438.0	1752.1	438.0	1752.1
TS	499.5	1998.1	499.5	1998.1	999.0	1998.1	499.5	1998.1
2MTS	473.0	1892.1	473.0	1892.1	473.0	1892.1	473.0	1892.1
Positive control	1.95	nt	1.95	nt	0.977	nt	0.488	nt
	VAN		VAN		NOR		NOR	

VAN: vancomycin; NOR: norfloxacin; NYS: nystatin; nt: not tested. Data are the median values of at least three replicates.
